# Lagging behind by doing good: How volunteering prolongs unemployment

**DOI:** 10.1111/1468-4446.13182

**Published:** 2025-01-05

**Authors:** Elisabeth Lilleøre Holstein, Hans‐Peter Y. Qvist

**Affiliations:** ^1^ Department of Sociology and Social Work Aalborg University Aalborg Denmark

**Keywords:** event history analysis, unemployment, volunteering

## Abstract

This study contributes new insights into whether volunteering improves the employment prospects of jobless individuals by examining its relationship with the speed at which they secure new jobs— an outcome that has received limited attention in previous research. Our comprehensive data enables us to investigate this by constructing an event history dataset that merges information from the Danish Volunteer Survey with administrative register data. Our results show that when we adjust for variations in education and labor market experience, jobless individuals who volunteer remain unemployed approximately two weeks or 31 percent longer than those who do not. Although our results remain correlational, they challenge the wisdom of promoting volunteering as a reemployment strategy, which some governments in European countries already do while others consider doing so. We recommend that policymakers reconsider the promotion of volunteering as a reemployment tool and call for further research into the relationship between volunteering and unemployment duration, particularly in different national contexts.

## 1 INTRODUCTION

Sociologists are engaged in an ongoing discussion about how volunteering affects jobless individuals' employment prospects. On the one side, optimists argue that volunteering offers jobless individuals opportunities to develop skills and establish social networks, bolstering their labor market prospects (Spera et al., 2015). In contrast, skeptics point to empirical evidence that challenges these claims and suggests that jobless individuals could use their time and energy more effectively by pursuing education or job training instead of volunteering (Petrovski et al., 2017).

While sociologists continue to debate these issues, several European governments have already embraced volunteering as an employment strategy for jobless individuals. The United Kingdom (UK) has been at the forefront of this movement, with its government promoting volunteering as an employment strategy for over a decade (Kamerāde & Paine, 2014; Paine et al., 2013). This message has resonated with the public, as demonstrated by a recent survey conducted among recent volunteers in the UK (Hogg & Smith, 2021). According to the survey, 15% of these volunteers expressed that they started volunteering to improve their chances of getting a new or better job. In addition, 58% somewhat or strongly agreed that volunteering has improved their job prospects.

The notion that volunteering can boost participants' job prospects, while prominently highlighted in the UK, is also gaining traction in other European countries, including Denmark. In Denmark, evidence suggests that roughly one‐third of all volunteers believe that volunteering gives an advantage in job searches (Espersen et al., 2021). Moreover, a recent study shows that unemployed individuals in Denmark rank job‐related factors, such as gaining practical experience, as a more important reason for volunteering than employed individuals (Holstein et al., 2023).

However, there is a need for more robust empirical evidence to determine whether volunteering improves jobless individuals' employment prospects in order to justify these government initiatives. Fortunately, as a critical step in this regard, sociologists have recently taken advantage of the expanded availability of longitudinal data, enabling them to investigate how volunteering relates to jobless individuals' future employment prospects. This development has significantly improved the state of research on the topic, which previously solely comprised cross‐sectional studies that did not shed light on the cause‐and‐effect relationship between volunteering and job prospects.

Nevertheless, the effect of volunteering on jobless individuals' employment prospects remains uncertain due to conflicting findings. While some longitudinal studies, such as Spera et al. (2015) and Paine et al. (2013), suggest that volunteering improves jobless individuals' employment prospects, other studies have found no significant effects or adverse effects under certain circumstances (Mao & Normand, 2022; Paine et al., 2013; Petrovski et al., 2017). Notably, the results appear to depend on the methodology used in the studies. Among the longitudinal studies, those employing methodologies addressing unobserved heterogeneity, such as fixed effects or instrumental variable regression, tend to provide evidence against the employment advantages of volunteering (Mao & Normand, 2022; Petrovski et al., 2017). This tendency is probably because these studies can better isolate the impact of volunteering from other factors that influence employment prospects. Additionally, existing evidence suggests a strong link between individuals' employment status, their likelihood of volunteering, and future employment prospect (Gangl, 2006; Nilsen & Reiso, 2011; Piatak, 2016; Wiertz & Lim, 2019; Wilson & Musick, 1997). This close relationship between prior employment, volunteering, and future job prospects complicates the study of how volunteering impacts employment prospects, as individuals may choose to volunteer based on their current employment status, which in turn affects their future job opportunities.

Although existing longitudinal studies have examined the relationship between volunteering and the likelihood of future employment for jobless individuals, several aspects of their job prospects remain unexplored. Notably, due to limitations in available data, researchers have concentrated on how volunteering ultimately impacts the transition from unemployment to employment but have not investigated its impact on the duration of unemployment experienced by individuals. Knowledge about this impact is important because, in contemporary societies characterized by fast‐evolving labor markets and frequent job changes due to technological advancements, the speed at which people can find new jobs after being laid off is crucial for ensuring dynamic and adaptable labor markets.

Against this backdrop, we present fresh evidence regarding the relationship between volunteering and jobless individuals' employment prospects, focusing on the time individuals spend unemployed before securing a job. To achieve this, we construct an event‐history dataset by merging data from the Danish Volunteer Survey collected in 2017 with weekly information about the individuals' labor market statuses from administrative registers. Using these data, we make two contributions to the literature.

First, we assess how volunteering relates to the duration of unemployment using time‐to‐event models rather than solely assessing whether individuals secure new employment by a predetermined follow‐up date, as is typically done in traditional panel studies. Moreover, we use the rich data from Danish administrative registers to precisely adjust for individuals' education and work experience. Notably, we control for individuals' actual work experience rather than just potential labor market experience, which researchers often use as a proxy when a measure for actual labor market experience is unavailable. This precise adjustment is important because volunteers tend to have higher levels of education and more labor market experience than non‐volunteers. As a result, they can secure new jobs more rapidly, regardless of their involvement in volunteering, making it important to adjust for these factors adequately.

Second, we explore how the relationship between volunteering and unemployment duration for jobless individuals depend on their intensity of involvement in volunteering. Specifically, we examine whether the strength of the relationship varies by the number of hours spent on volunteer activities. To do this, we use the detailed information from the Danish Volunteer Survey on the number of hours spent volunteering within the last year.

## 2 THEORETICAL BACKGROUND AND HYPOTHESES

Sociologists have proposed conflicting theories about how volunteering affects the employment prospects of jobless individuals. Some theories argue that volunteering improves jobless individuals' employment prospects by providing them with various resources. In contrast, other theories suggest that volunteering might delay the job search process by diverting participants' attention away from it. The following sections discuss these theoretical understandings separately and derive a set of competing hypotheses.

### 2.1 How volunteering might improve jobless people's employment prospects

To better understand theories proposing that volunteering can improve jobless people's employment prospects, it is helpful to discern between *participation* and *signaling effects*. Within this categorization, participation effects pertain to acquiring resources, such as human and social capital, that enhance volunteers' capability to secure employment. In contrast, signaling effects occur when volunteers enhance their employment prospects by conveying a positive signal to potential employers, thereby improving these employers' perception of their labor market value (Qvist & Munk, 2018).

A central way jobless people can improve their employment prospects is to enhance their human capital, referring to skills, qualifications, and competencies (Becker, 2006). The traditional and most effective means of human capital acquisition are education and labor market experience (Becker, 2006; Mincer, 1958, 1974). Nevertheless, evidence indicates that people can acquire human capital from diverse sources beyond these traditional ones. Due to the similarities between volunteering and paid employment, scholars have, for example, argued that voluntary organizations could function as informal training grounds, enabling people to cultivate labor‐market‐relevant skills and competencies (Benenson & Stagg, 2016; Musick & Wilson, 2008; Nichols & Ralston, 2011).

In connection with the above, extensive research has been conducted to unveil the various skills and competencies that individuals can develop through volunteering (Benenson & Stagg, 2016; Brady et al., 1995; Handy & Greenspan, 2009; Musick & Wilson, 2008; Nichols & Ralston, 2011). These studies propose, for instance, that individuals who volunteer, particularly in roles involving board responsibilities or the like, can enhance their reading, writing, fundraising, newsletter editing, and presentation skills, which are valuable assets in many jobs (Brady et al., 1995). Additionally, scholars have argued that volunteers sometimes acquire or develop specialized skills that are valuable in specific niches in the labor market. For example, volunteers in emergency rescue squads or volunteer firefighters develop skills they can use professionally in paid jobs with similar tasks (Musick & Wilson, 2008).

Beyond skill development, volunteering may improve the employment prospects of jobless individuals by strengthening their social networks, either by increasing the number of connections they have or enhancing the quality of their networks. As Granovetter (1973) noted early on, the number of social connections an individual has can be crucial during a job search. Weak ties, such as acquaintances, connect individuals to a wider network beyond their immediate social circles and can provide access to crucial information about job openings. However, the effectiveness of social networks in a job search depends not just on their size but also on their quality. Connections to influential individuals can offer significant advantages in accessing job opportunities in the labor market (Lin, 1999; Lin & Dumin, 1986; Lin et al., 1981). While evidence supports that volunteering can improve both the size and quality of individuals' social networks, the impact appears modest when accounting for the fact that people who are already well‐connected are more likely to volunteer in the first place (Benton, 2016; Dederichs, 2024; Van Ingen & Kalmijn, 2010).

In addition to human and social capital enhancement, scholars have argued that people may send a powerful signal to potential employers by volunteering. In general, signaling theory emphasizes that hiring managers often rely on clues to access potential candidates because they do not have perfect information about their levels of human capital (Spence, 1973). In the context of volunteering, signaling theory highlights that by engaging in volunteer work, jobless people send a positive signal to potential employers of being motivated, productive, and compassionate (Wallrodt & Thieme, 2020). It may also signal desirable personal qualities, such as dedication, drive, emotional stability, extraversion, and a cooperative attitude. Thus, jobless people may signal their personal qualities through volunteering, which can be decisive in cases where they lack education and experience (Day & Devlin, 1998; Ziemek, 2006).

In summary, volunteering may reduce the time jobless people are unemployed. This pattern will likely occur if volunteers accumulate resources through participation and send positive signals to potential employers about their skill set, work ethic, and personality. Consequently, our first hypothesis is:

H 1 Jobless people who volunteer experience shorter unemployment durations than non‐volunteers.

Furthermore, if volunteering brings advantages to those who participate, it is reasonable to think that individuals who volunteer more frequently will benefit more. Consequently, we propose an additional complementary hypothesis:

H 1a Jobless people who dedicate much time to volunteering experience shorter unemployment durations than those who volunteer less or not at all.

### 2.2 How volunteering might worsen jobless people's employment prospects

The existing research also suggests that volunteering might adversely affect jobless people's employment prospects (Mao & Normand, 2022; Paine et al., 2013; Petrovski et al., 2017). In labor market research, scholars typically use the term “lock‐in effects" to refer to scenarios where individuals who participate in active labor market programs (ALMPs), such as job training programs or wage subsidies, become “locked in” to these programs because they face disincentives to exit unemployment and seek regular employment (Lammers & Kok, 2021). We consider it likely that similar effects could arise from volunteering. In this context, we discern two distinct forms of disincentives that might arise from volunteering: *Time and energy constraints* and *emotional attachment*.

Volunteering requires a significant investment of time and energy, and studies indicate that it can detract from time that could be spent on paid employment (Qvist, 2021). As a result, volunteering can likely take time and energy away from jobless people's job search, prolonging unemployment. The extensive literature in labor market economics that evaluates ALMPs suggests that such programs frequently produce lock‐in effects, at least in the short run, because jobless people's job search efforts decrease or cease during their program participation (Fremigacci & Terracol, 2013; Kyyrä et al., 2013; Lammers & Kok, 2021; Vooren et al., 2019).

Although ALMPs may initially produce lock‐in effects, effective programs eventually produce participation effects, leading to long‐term positive outcomes (Lammers & Kok, 2021). However, Lammers and Kok (2021) describe three situations where lock‐in effects can remain counterproductive. The first is a situation where the jobless person has skills and personal characteristics that would readily allow him or her to secure employment *without* participating in a program. The second is a situation where the program runs very long. The third is a situation where ALMPs remain in place during an economic boom where all people's chances of finding work are significantly better than during more challenging economic times (Lammers & Kok, 2021).

In the context of volunteering, it is noteworthy that many volunteers are capable individuals who could likely find paid employment. Additionally, volunteer positions often do not have a specific end date and leaving them can be challenging due to social pressure. For these reasons, such volunteer activities could produce short‐term lock‐in effects and possibly remain counterproductive in the long run.

In addition to being time and energy‐consuming, volunteers frequently become emotionally attached to their organizations and other participants. These bonds might make volunteers less motivated to seek paid employment if they are sufficient to meet their emotional needs. In this context, research suggests that volunteering can fulfill various psychological needs, such as feeling accepted and socially included (Clary et al., 1998). Consequently, the sense of belonging and community people may find in volunteering might become so significant that it takes the place of the fulfillment they would otherwise seek from paid work (Henriksen et al., 2023).

Additionally, volunteering might offer stability and purpose for marginalized individuals, possibly leading them to temporarily put off their job search. For example, volunteer organizations can be places where unemployed people find solidarity with others facing similar challenges, such as economic hardship or disability (Baines & Hardill, 2008; Cohen, 2009). These connections can be crucial for managing and coping with their situations. This mechanism is likely because volunteer organizations offer more inclusive social environments than many workplaces. Unlike workplaces, which often operate within hierarchical, competitive, or profit‐driven frameworks, volunteer organizations are typically mission‐driven and foster collaborative environments that prioritize participation and community building (Musick & Wilson, 2008; Putnam, 2000). As a result, marginalized individuals may perceive these as “safe spaces” and might be reluctant to give this up to seek paid employment.

In summary, volunteering may prolong the time jobless people are unemployed. This pattern is likely to occur if volunteering produces lock‐in effects, which we propose can arise because volunteering is *time‐ and energy‐consuming* and can create an *emotional attachment* that is challenging to let go. Consequently, our second hypothesis is:

H 2 Jobless people who volunteer experience longer unemployment durations than non‐volunteers.

Moreover, if volunteering produces lock‐in effects, it is reasonable to expect individuals who volunteer more frequently to experience more prolonged unemployment. Consequently, we propose an additional hypothesis:

H 2a Jobless people who dedicate much time to volunteering experience longer unemployment durations than those who volunteer less or not at all.

## 3 DATA, MEASURES, AND ANALYTICAL STRATEGY

We use data from the Danish Volunteer Survey collected in 2017. The private company, Rambøll Management Consulting, collected the survey, representing the general Danish population aged 16–86. The survey included 4.977 respondents and had a response rate of 42.1%.

A vital feature of the survey that enhances its value for our specific objectives is that it implemented an approach to maximize the response rates of social security recipients. This approach involved an initial screening question sent to everyone in the sample to determine their employment status. This screening procedure allowed the survey team to focus their follow‐up efforts on encouraging responses from those not currently in the labor market, a group often missed in surveys (Rambøll Management Consulting, 2017).

We merged these survey data with various administrative registers to examine how volunteering relates to unemployment duration. The merging of survey and administrative register data is possible in Denmark through a unique personal identification number that all residents in Denmark must have, and it presents unique opportunities to study the possible labor market benefits of volunteering (Qvist, 2022). In compliance with the rules and regulations of Statistics Denmark and the Danish Data Protection Agency, we carried out all data management and analysis via remote access to servers located at Statistics Denmark.

The most important source of information is the Danish Register for Evaluation of Marginalization (DREAM). This register contains weekly information on public transfer incomes, unemployment registrations, and participation in ALMPs for individuals who receive public transfer incomes. However, we draw on several other registers to obtain socioeconomic and demographic information about all respondents, for example, education, labor market experience, and country of origin.

Our analytic sample includes individuals who experienced unemployment at some point in 2018, which is the year right after they took part in the survey. We identified these individuals based on information about unemployment benefits and social security transfers from DREAM. Most of this group received unemployment benefits or financial assistance, while a minor proportion received different kinds of education allowance, and a few immigrants received special financial assistance, referred to as “integration” allowance.

It is important to note that our study focuses on unemployed individuals while excluding those outside the workforce. Danish caseworkers must classify financial assistance recipients as “job‐ready” or “activity‐ready”. Job‐ready financial assistance recipients are those classified as part of the labor force without jobs. These individuals have to seek employment to remain actively in the benefit scheme. In contrast, activity‐ready financial assistance recipients are not considered part of the workforce due to various circumstances, notably health issues (Ravn & Nielsen, 2019). We additionally exclude young people who receive state education grants, people in flexi‐jobs, and people on early retirement benefits who are not considered part of the workforce.^1^

Ultimately, our sample consists of 442 individuals who were unemployed at some point in 2018. Of these, 437 were included in the analytical sample after excluding individuals with missing data on any variable. In the analyses, we follow these individuals for 3 years, allowing them to experience unemployment multiple times. We observed 1027 unemployment spells from the 437 individuals during the three‐year period. Out of the 437 individuals, 174 experienced only one unemployment spell, while 263 experienced two or more.

### 3.1 Dependent variable

The outcome variable is the duration of unemployment in weeks during the three‐year period from 2018 to 2020^2^. To code the variable, we first recorded the week a respondent in the analytic sample began receiving unemployment benefits in 2018. Next, we counted the weeks that passed before the respondent no longer received unemployment benefits, the observation period ended, or the individual left the study for unrelated reasons (right censoring). Each unemployment spell is treated as a separate observation, meaning that if an individual experienced multiple unemployment spells during the 3‐year period, each spell is recorded as a distinct observation in the dataset. However, we treated gaps in the unemployment spell under 4 weeks as continuous benefit receipts.

### 3.2 Independent variable

The independent variable is binary and indicates whether the respondent has volunteered in 2017 based on the survey data. The survey asked respondents if they had volunteered in one or more of a listed set of different areas within the previous year. The different areas corresponded to the International Classification of Nonprofit Organizations (Salamon & Anheier, 1992). In the sample of 437 individuals, 135 volunteered in 2017, equivalent to about 31%.

### 3.3 Control variables

A substantial body of literature documents that volunteers exhibit disproportionate resourcefulness regarding socioeconomic status, health, and social networks (Ma & Konrath, 2018). These resource factors are also related to unemployed people's employment prospects, which means they can confound the relationship between volunteering and employment prospects.

Consequently, to estimate the effect of volunteering on unemployment durations, the above factors must be sufficiently controlled for in statistical models. Our control variables include educational level, labor market experience, type of unemployment benefit, unemployment history, self‐rated health, gender, ethnicity, and age.

Educational level is based on administrative registers' information originating from educational institutions' reporting, based on International Standard Classification of Education (ISCED). The variable was coded in three levels as follows: level 1 = Short or upper secondary education (including vocational or general upper secondary programs), level 2 = Medium‐cycle or bachelor‐level education (combined), and level 3 = Long‐cycle higher education or PhD.

Labor market experience is years of labor market experience prior to 2017. The measure is an approximation based on mandatory pension payments. It is important to note that this variable directly captures actual labor market experience, in contrast to the proxy variables used in most studies (Miller, 1993). The labor market experience variable, provided by Statistics Denmark, ranges from 0 to 1000. Within this scale, a total score of 750 corresponds to one year of part‐time employment, while 1000 represents one year of full‐time employment. We rescaled this variable by dividing it by 1000. Consequently, five on this rescaled measurement equates 5 years of full‐time work experience.

The type of unemployment benefit is based on information from the DREAM register. It is an indicator variable distinguishing individuals who receive unemployment benefits from everyone else.

The variable measuring an individual's recent unemployment history is continuous, ranging from 0 to 3. The variable reflects one's unemployment rate over the 3 years leading up to 2017. A score of 0 indicates that the individual was not unemployed at all during this period, while a score of 3 represents continuous unemployment throughout the entire 3‐year timeframe. Scores between 0 and 3 capture varying levels of unemployment during this period.

Health is a self‐rated measure based on the Danish Volunteer Survey, in which the respondents were asked about their general health. The response categories are (5) Very good, (4) Good, (3) Reasonably, (2) Bad and (1) Very Bad.

Table 1 presents descriptive statistics for all variables included in the analysis.

**TABLE 1:** Descriptive statistics.

	Mean	Standard deviation
Unemployment duration	13.96	21.19
Volunteer (past year)	0.31	0.46
Volunteer hours past month		
No hours	0.74	0.44
1–19 h	0.20	0.40
20 or more	0.06	0.23
Unemployment benefits	0.80	0.40
Labor market experience	11.40	10.48
Unemployment history	1.00	1.17
Educational level		
Low	0.56	0.50
Medium	0.33	0.47
High	0.12	0.32
Self‐rated health (1–5)	4.03	0.85
Female	0.62	0.49
Immigrant background	0.11	0.31
Age	34.25	11.89
Observations	1027	

## 4 ANALYTICAL STRATEGY

We employ lognormal accelerated failure time (AFT) models with a shared frailty term to estimate how volunteering relates to unemployment duration while accounting for right‐censoring. The frailty term is necessary because we include multiple unemployment spells from the same individuals. The term captures time‐constant individual traits—such as personality or job search motivation—that are not covered by our covariates but influence the duration of unemployment.^3^

In our AFT models, the dependent variable is the natural logarithm of unemployment durations, meaning that their coefficients directly reveal how much shorter or longer unemployment durations volunteers experience compared to non‐volunteers. Specifically, a negative coefficient for the volunteering variable indicates that jobless individuals who volunteer experience shorter unemployment durations than non‐volunteers. Conversely, a positive coefficient indicates that jobless individuals who volunteer experience longer unemployment durations. These coefficients, therefore, offer a straightforward means of appraising our hypotheses.

More formally, our AFT model with a shared frailty term can be expressed as follows:

$$\log\left(t_{\text{ij}}\right)=\alpha +\beta_{1}V_{i}+\beta_{X}X_{i}+u_{i}+\varepsilon_{\text{ij}}$$

Here $\log\;\left(t_{\text{ij}}\right)$ is the natural logarithm of the weekly duration of unemployment spell *j* experienced by the individual *i*,^4^ α is a constant, $V_{i}$ is an indicator of volunteering, $X_{i}$ is a vector of the control variables, $u_{I}$ are the shared frailty term that vary across individuals, and $\varepsilon_{ij}$ is an error term. The years of labor market experience are included along with a squared term to address the possibility that the effect of each additional year of experience diminishes over time. Since we use lognormal AFT models, the error term is assumed to follow a standard normal distribution. To ensure the robustness of our results, we also fitted semi‐parametric Cox regression models, which relax this distributional assumption (see Tables S2a and S3a). These models yield results largely consistent with those in the primary analysis.^5^

In our analyses, we focus on $\beta_{1}$ as the critical factor of interest because it reveals how volunteering relates to jobless people's unemployment durations. When we exponentiate this coefficient, it expresses in percentage terms how much shorter or longer unemployment durations that jobless people who volunteer experience unemployment durations compared to non‐volunteers.

## 5 RESULTS

We begin this section by looking at the relationship between volunteering and unemployment durations. Next, we consider how this relationship varies by the amount of time invested in volunteering.

## 6 VOLUNTEERING AND UNEMPLOYMENT DURATION

Table 2 presents the results of our lognormal AFT models. Model 1 presents estimates of the relationship between volunteering and unemployment duration, excluding the frailty term and without controlling for any covariates. In this simple model, we observe that the coefficient for volunteering is positive, indicating that volunteers tend to experience longer periods of unemployment than non‐volunteers; however, the coefficient is statistically insignificant (*β* = 0.096, *p* > 0.05).

**TABLE 2:** Lognormal accelerated failure time models predicting unemployment duration by volunteering.

	Model 1	Model 2	Model 3
Volunteer	0.096	0.321**	0.272*
	(0.130)	(0.118)	(0.127)
Unemployment benefits		−2.161***	−2.203***
		(0.148)	(0.148)
Labor market experience		−0.047*	−0.041
		(0.020)	(0.022)
Labor market experience × labor market experience		0.001**	0.001*
		(0.000)	(0.001)
Unemployment history		0.029	0.004
		(0.049)	(0.055)
Educational level (ref. = low)			
Medium		−0.145	−0.111
		(0.126)	(0.136)
High		−0.472**	−0.491*
		(0.183)	(0.203)
Self‐rated health (1–5)		−0.155*	−0.131
		(0.069)	(0.075)
Female		−0.074	−0.088
		(0.112)	(0.123)
Immigrant background		−0.288	−0.196
		(0.179)	(0.191)
Age		−0.002	−0.001
		(0.009)	(0.009)
Constant	1.265***	4.083***	3.965***
	(0.074)	(0.352)	(0.370)
Ln(σ)	0.400***	0.269***	0.165***
	(0.027)	(0.027)	(0.033)
Ln(θ)			−1.310***
			(0.198)
Observations	1027	1027	1027

*Note*: Standard errors in parentheses.

*p < 0.05, **p < 0.01, ***p < 0.001.

Next, in Model 2, we present estimates of the relationship between volunteering and unemployment duration. Like Model 1, the frailty term is excluded, but now we include the control variables to address potential confounding factors. Compared to Model 1, we observe that the coefficient for volunteering in Model 2 remains positive, but it is noticeably larger in magnitude and statistically significant (*β* = 0.321, *p* < 0.01). This finding suggests that the association between volunteering and longer unemployment durations becomes more pronounced when we control for factors such as educational levels and labor market experience. To clarify, when we observe a small and insignificant coefficient for volunteering in Model 1, it is because education and labor market experience suppress the association between volunteering on unemployment durations. This suppression occurs because education and labor market experience make people more inclined to volunteer and reduce their unemployment durations. As a result, the relationship between volunteering and jobless people's unemployment durations is smaller than its actual magnitude when these factors are not controlled for.

Lastly, in Model 3, we present estimates of the relationship between volunteering and unemployment duration, incorporating both the control variables and the frailty term, which adjust for unmeasured individual differences shaping unemployment durations. The coefficient for the volunteering variable in this model, while slightly smaller in magnitude compared to Model 2, which excluded the frailty terms, remains positive and statistically significant (0.272, *p* < 0.05). The size of the coefficient indicates that volunteers, all other things being equal, experience approximately 31% longer unemployment durations than non‐volunteers ((exp (0.272) − 1) × 100 ≈ 31%).

**FIGURE 1:**
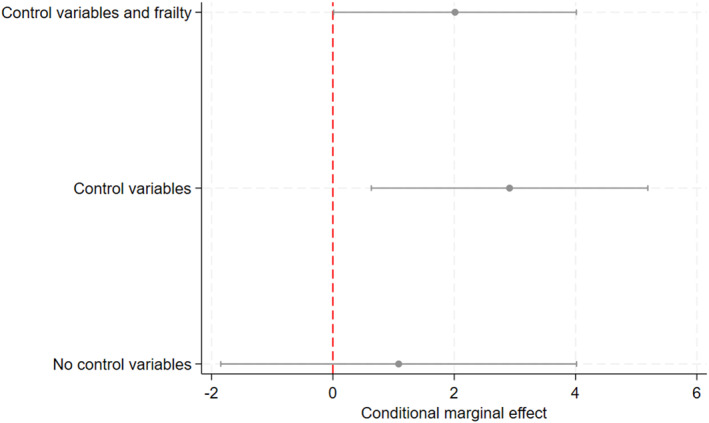
Conditional marginal effects of volunteering on unemployment durations in weeks and calculated with respect to the predicted mean of unemployment durations.

To clarify the strength of the relationship, we also present conditional marginal effects based on predictions of the mean unemployment durations when we set all other factors to their average values. Based on Model 3, the predicted mean unemployment duration is 8.45 weeks for volunteers and 6.44 weeks for non‐volunteers. The conditional marginal effect, or the difference between these two means, is 2.01 (or 31%), suggesting that that jobless people who volunteer remain unemployed for approximately two weeks longer than those who do not, as also shown in Figure 1.^6^ Additionally, the Figure shows that the conditional marginal effect is significant only when control variables are included, as education and labor market experience suppress the relationship between volunteering and unemployment duration.

To explore how the relationship between volunteering and unemployment durations differs based on the types of benefits individuals receive, as well as their level of education and age, we extended the AFT models to include interaction terms. However, the interaction terms proved insignificant (results are available in Table S4a). Consequently, we find no evidence indicating that the relationship between volunteering and unemployment duration differs substantially depending on the socioeconomic factors we consider.

## 7 ARE THE LOCK‐IN EFFECTS MORE PRONOUNCED FOR HIGH‐INTENSITY VOLUNTEERS?

In this section, we examine how the relationship between volunteering and unemployment duration varies by how much time jobless people invest in volunteering activities. Specifically, we consider whether the relationship is stronger for those who spend 20 h per month or more than those who spend less or no time.

Table 3 presents lognormal AFT models with shared frailty terms that predict unemployment duration by volunteering intensity. The point estimates indicate that jobless individuals who spend much time on volunteering activities experience longer unemployment durations than those who spend less or no time. However, it is important to note that the coefficients lack statistical significance. This lack of significance is likely due to the relatively small number of individuals volunteering more than 20 hours per month, leading to low statistical power in the analysis.^7^

**TABLE 3:** Lognormal accelerated failure time models predicting unemployment duration by volunteering intensity.

	Model 1	Model 2
Monthly hours of volunteering (ref. = No volunteering)		
1–19 h	0.148	0.137
	(0.136)	(0.149)
20 h or more	0.298	0.330
	(0.236)	(0.254)
Unemployment benefits	−2.162***	−2.203***
	(0.148)	(0.148)
Labor market experience	−0.046*	−0.041
	(0.021)	(0.023)
Labor market experience × labor market experience	0.001**	0.001*
	(0.000)	(0.001)
Unemployment history	0.026	−0.000
	(0.050)	(0.055)
Educational level (ref. = low)		
Medium	−0.117	−0.099
	(0.125)	(0.136)
High	−0.449*	−0.482*
	(0.183)	(0.204)
Self‐rated health (1–5)	−0.141*	−0.120
	(0.069)	(0.075)
Female	−0.079	−0.092
	(0.113)	(0.123)
Immigrant background	−0.292	−0.191
	(0.180)	(0.192)
Age	−0.002	−0.001
	(0.009)	(0.009)
Constant	4.078***	3.949***
	(0.352)	(0.370)
Ln(σ)	0.271***	0.163***
	(0.027)	(0.033)
Ln(θ)		−1.279***
		(0.194)
Observations	1027	1027

*Note*: Standard errors in parentheses.

*p < 0.05, **p < 0.01, ***p < 0.001.

## 8 DISCUSSION AND CONCLUSION

Sociologists are currently engaged in a discussion about the effectiveness of volunteering in improving the employment prospects of jobless individuals. This scientific discussion is highly relevant to policy because several governments in Europe, notably the UK, promote volunteering as an employment strategy for jobless individuals, with governments in other countries considering the adoption of similar initiatives.

Our study contributes to this discussion by providing evidence on how volunteering relates to jobless individuals' unemployment durations. To achieve this, we used lognormal AFT models with a shared frailty term to analyze time‐to‐event data created by merging the Danish Volunteer Survey with administrative records about individuals' employment status.

Our findings reveal a significant positive relationship between volunteering and unemployment duration. Point estimates suggest that, on average, jobless individuals who volunteer, all else being equal, remain unemployed approximately two weeks, or 31%, longer than non‐volunteers. Furthermore, we find no evidence that this relationship varies significantly across educational levels, labor market experience, or age.

While our evidence remains correlational, as it is based on observational data, it supports the theory that volunteering can prolong unemployment for jobless individuals due to a lock‐in effect. Although unobserved confounding cannot entirely be ruled out in an observational study, we control for the most critical potential confounders, such as education, actual labor market experience, and individuals' unemployment history, reducing the risk of bias from unobserved factors.

However, certain factors that may influence both volunteering and unemployment duration, such as deeply ingrained personality traits, are difficult to control for, and others, like religious affiliation, are missing from our data. However, we control for immigration status, which covers most of the variation in religious affiliation in Denmark, where religious differences are mainly found among immigrant groups. Still, this variable may not fully capture the potential influence of religious affiliation.

Another limitation is that we cannot determine the exact timing of volunteering. We only know whether individuals volunteered within the past year at the time of the survey, making it impossible to distinguish those who started volunteering after becoming unemployed from those who had already been volunteering. This is important, as the latter group may not use volunteering as an employment strategy. Additionally, some unemployed individuals may already know they will start a new job, so for them, volunteering neither helps nor delays reemployment. Finally, the potential benefits of volunteering—such as building human and social capital or its signaling effects—may diminish over time. This suggests that volunteering at the time of the survey is more likely to impact reemployment prospects earlier in the observation window than later. Ideally, future research should track individuals' volunteering and employment statuses at multiple points in time to address these timing‐related issues more effectively.

Given these methodological limitations, we recommend further research to determine whether the relationship we observed reflects a true causal lock‐in effect of volunteering on the unemployment duration of jobless individuals. Future research could use causal inference methods, such as identifying an instrumental variable to isolate exogenous variation in volunteering and estimate its causal impact. Moreover, if volunteering does create lock‐in effects, the mechanisms behind it—such as time constraints or emotional attachment—remain unexplored.

Although the causal nature of the relationship remains uncertain, we advise policymakers in Denmark and elsewhere to reconsider promoting volunteering as a reemployment strategy for jobless individuals, as has been done in the UK for several years. Our study adds to growing evidence suggesting that volunteering is, at best, an ineffective employment strategy for jobless individuals and, at worst, potentially counterproductive—at least in Denmark (Petrovski et al., 2017). However, volunteering may still impact the type of job individuals secure when reentering the labor market. Evidence from other studies shows that volunteering can improve job quality, as reflected in occupational status (Ruiter & de Graaf, 2009) and higher earnings among graduates (Qvist & Munk, 2018). These job‐quality benefits might be more substantial for job individuals, but this warrants further research. Additionally, promoting volunteering among the unemployed might be valuable for reasons beyond employment, such as maintaining mental health during unemployment.

It is also important to acknowledge that findings from Denmark differ from previous studies in the UK and US, which found positive relationships between volunteering and reemployment prospects (Paine et al., 2013; Spera et al., 2015). Unfortunately, our current understanding of these cross‐national differences is limited by the lack of comparative studies and the varying methodologies used in these single country studies. As the field evolves, future research should explore how the relation between volunteering and employment prospects varies across welfare regimes, considering that the feasibility of substituting job searching with volunteering may be higher in countries where unemployment benefits support a higher standard of living (Gil‐Lacruz & Marcuello, 2013). This would provide deeper insights into the link between volunteering and labor market reintegration.

## CONFLICT OF INTEREST STATEMENT

The authors declare no conflicts of interest.

## Supporting information

Supporting Information S1

## Data Availability

The 2017 Danish Volunteer Survey is not publicly available. However, researchers interested in these data can request them from the Danish Ministry of Social Affairs, Housing, and Senior Citizens. The register data are not publicly available either. Individual‐level register data in Denmark are only made available to authorized researchers upon approval from Statistics Denmark. Researchers who want to replicate our results must be granted access to the relevant data by Statistics Denmark.

## ENDNOTES

^1^
In Denmark, unemployment benefits are a state‐subsidized insurance scheme against unemployment. It is possible to receive unemployment benefits for 2 years. For an individual to meet the eligibility criteria for receiving unemployment benefits, the individual must be registered as a job seeker, available for the labor market, have been a member of an unemployment insurance fund (“a‐kasse”) for at least 1 year, have been employed as a wage earner for at least 52 weeks in the last 3 years (for fully insured individuals) and 34 weeks in the last 3 years (for partially insured individuals), or have been significantly self‐employed during a similar period. If an individual exhausts their entitlement to unemployment benefits, there is an option, under certain conditions, to receive financial assistance (“kontanthjælp”) from the municipality.

^2^
Although our observation window includes the year 2020, when the Covid‐19 pandemic occurred, nearly all re‐employment events took place beforehand, resulting in minimal impact on our findings

^3^
To ensure the robustness of our findings, we estimated AFT models that consider only each individual's first experience of unemployment. These results indicate that the relationship between volunteering and unemployment duration is, if anything, stronger than what we present in our primary findings (see Table S1a).

^4^
To handle ties in the observed unemployment durations (i.e., unemployment durations of the same length), we subtracted a tiny random value from each observation. While this solution originally proposed by Hosmer and Lemeshow (1999) is not ideal, evidence suggests it performs well in practice (Borucka, 2014).

^5^
While the AFT model allows for a direct interpretation of time ratios—indicating how much longer or shorter individuals remain unemployed due to volunteering—the Cox model provides hazard ratios, which reflect the likelihood of re‐employment relative to volunteering status. The Cox model, being semi‐parametric, does not make assumptions about the functional form of the baseline hazard, whereas the AFT model requires specifying a distribution for the time‐to‐event data. Despite these differences in model assumptions, we found that both the AFT and Cox models yielded similar overall conclusions: The AFT model estimated that volunteering was associated with a 31 percent increase in unemployment duration, while the corresponding hazard ratio from the Cox model indicated a similar 28 percent increase.

^6^
The conditional marginal effect can be calculated with respect to the median instead of the mean. Figure S1A in the online supplementary material presents conditional marginal effects based on predictions of the median unemployment duration when we set all other factors to their average values. The conditional marginal effect based on the predicted median unemployment duration for jobless people who volunteer is approximately 1 week longer than for those who do not.

^7^
Adjusting the cut‐off points for high‐intensity volunteering, whether by lowering or raising the threshold, do not affect the direction or significance of our estimates.

## References

[bjos13182-bib-0001] Baines, S. , & Hardill, I. (2008). ‘At least I can do something’: The work of volunteering in a community beset by worklessness. Social Policy and Society, 7(3), 307–317. 10.1017/s1474746408004284

[bjos13182-bib-0002] Becker, G. S. (2006). Human capital: A theoretical and empirical analysis, with special reference to education (3rd ed.). The University of Chicago Press.

[bjos13182-bib-0003] Benenson, J. , & Stagg, A. (2016). An asset‐based approach to volunteering: Exploring benefits for low‐income volunteers. Nonprofit and Voluntary Sector Quarterly, 45(1), 131S–149S. 10.1177/0899764015604739

[bjos13182-bib-0004] Benton, R. A. (2016). Uniters or dividers? Voluntary organizations and social capital acquisition. Social Networks, 44, 209–218. 10.1016/j.socnet.2015.09.002

[bjos13182-bib-0005] Borucka, J. (2014). Extensions of cox model for non‐proportional hazards purpose. Ekonometria(45), 85–101. 10.15611/ekt.2014.3.07

[bjos13182-bib-0006] Brady, H. E. , Verba, S. , & Schlozman, K. L. (1995). Beyond SES: A resource model of political participation. American Political Science Review, 89(2), 271–294. 10.2307/2082425

[bjos13182-bib-0007] Clary, E. G. , Snyder, M. , Ridge, R. D. , Copeland, J. , Stukas, A. A. , Haugen, J. , & Miene, P. (1998). Understanding and assessing the motivations of volunteers: A functional approach. Journal of Personality and Social Psychology, 74(6), 1516–1530. 10.1037/0022-3514.74.6.1516 9654757

[bjos13182-bib-0008] Cohen, A. (2009). Welfare clients' volunteering as a means of empowerment. Nonprofit and Voluntary Sector Quarterly, 38(3), 522–534. 10.1177/0899764008320196

[bjos13182-bib-0009] Day, K. M. , & Devlin, R. A. (1998). The payoff to work without pay: Volunteer work as an investment in human capital. Canadian Journal of Economics / Revue Canadienne d'Economique, 31(5), 1179–1191. 10.2307/136465

[bjos13182-bib-0010] Dederichs, K. (2024). Join to connect? Voluntary involvement, social capital, and socioeconomic inequalities. Social Networks, 76, 42–50. 10.1016/j.socnet.2023.07.004

[bjos13182-bib-0011] Espersen, H. H. , Fridberg, T. , Andreasen, A. G. , & Brændgaard, N. W. (2021). Frivillighedsundersøgelsen 2020.

[bjos13182-bib-0012] Fremigacci, F. , & Terracol, A. (2013). Subsidized temporary jobs: Lock‐in and stepping stone effects. Applied Economics, 45(33), 4719–4732. 10.1080/00036846.2013.797644

[bjos13182-bib-0013] Gangl, M. (2006). Scar effects of unemployment: An assessment of institutional complementarities. American Sociological Review. American Sociological Review, 71(6), 986–1013. 10.1177/000312240607100606

[bjos13182-bib-0014] Gil‐Lacruz, A. I. , & Marcuello, C. (2013). Voluntary work in europe: Comparative analysis among countries and welfare systems. Social Indicators Research, 114(2), 371–382. 10.1007/s11205-012-0150-5

[bjos13182-bib-0015] Granovetter, M. S. (1973). The strength of weak ties. American Journal of Sociology, 78(6), 1360–1380. 10.1086/225469

[bjos13182-bib-0016] Handy, F. , & Greenspan, I. (2009). Immigrant volunteering: A stepping stone to integration? Nonprofit and Voluntary Sector Quarterly, 38(6), 956–982. 10.1177/0899764008324455

[bjos13182-bib-0017] Henriksen, L. S. , Qvist, H. Y. , & Holstein, E. L. (2023). Forbindelsesveje mellem arbejde og frivilligt arbejde. In Gør frivilligt arbejde samfundet bedre? (pp. 199–223). Nyt fra Samfundsvidenskaberne.

[bjos13182-bib-0018] Hogg, E. , & Smith, A. (2021). Kickstarting a new revolution. In Social mobility: Unleashing the power of volunteering. Royal Voluntary Service.

[bjos13182-bib-0019] Holstein, E. L. , Qvist, H. Y. , & Henriksen, L. S. (2023). Frivillighed blandt borgere på kanten af arbejdsmarkedet. In Gør frivilligt arbejde samfundet bedre? (pp. 121–140). Nyt fra Samfundsvidenskaberne.

[bjos13182-bib-0020] Hosmer, D. W. , & Lemeshow, S. (1999). Applied survival analysis: Regression modeling of time to event data. Wiley.

[bjos13182-bib-0021] Kamerāde, D. , & Paine, A. E. (2014). Volunteering and employability: Implications for policy and practice. Voluntary Sector Review, 5(2), 259–273. 10.1332/204080514X14013593888736

[bjos13182-bib-0022] Kyyrä, T. , Parrotta, P. , & Rosholm, M. (2013). The effect of receiving supplementary UI benefits on unemployment duration. Labour Economics, 21, 122–133. 10.1016/j.labeco.2013.02.002

[bjos13182-bib-0023] Lammers, M. , & Kok, L. (2021). Are active labor market policies (cost‐)effective in the long run? Evidence from The Netherlands. Empirical Economics, 60(4), 1719–1746. 10.1007/s00181-019-01812-3

[bjos13182-bib-0024] Lin, N. (1999). Social networks and status attainment. Annual Review of Sociology, 25(1), 467–487. 10.1146/annurev.soc.25.1.467

[bjos13182-bib-0025] Lin, N. , & Dumin, M. (1986). Access to occupations through social ties. Social Networks, 8(4), 365–385. 10.1016/0378-8733(86)90003-1

[bjos13182-bib-0026] Lin, N. , Ensel, W. M. , & Vaughn, J. C. (1981). Social resources and strength of ties: Structural factors in occupational status attainment. American Sociological Review, 46(4), 393–405. 10.2307/2095260

[bjos13182-bib-0027] Ma, J. , & Konrath, S. (2018). A century of nonprofit studies: Scaling the knowledge of the field. Voluntas (Manchester, England), 29(6), 1139–1158. 10.1007/s11266-018-00057-5

[bjos13182-bib-0028] Mao, L. , & Normand, C. (2022). The effect of volunteering on employment: Evidence from the Irish longitudinal study on ageing (TILDA). The Journal of the Economics of Ageing, 21, 100350. 10.1016/j.jeoa.2021.100350

[bjos13182-bib-0029] Miller, C. F. (1993). Actual experience, potential experience or age, and labor force participation by married women. Atlantic Economic Journal, 21(4), 60–66. 10.1007/BF02302329

[bjos13182-bib-0030] Mincer, J. (1958). Investment in human capital and personal income distribution. Journal of Political Economy, 66(4), 281–302. 10.1086/258055

[bjos13182-bib-0031] Mincer, J. (1974). Schooling, experience, and earnings. Columbia University Press.

[bjos13182-bib-0032] Musick, M. A. , & Wilson, J. (2008). Volunteers a social profile. Indiana University Press.

[bjos13182-bib-0033] Nichols, G. , & Ralston, R. (2011). Social inclusion through volunteering: The legacy potential of the 2012 olympic games. Sociology, 45(5), 900–914. 10.1177/0038038511413413

[bjos13182-bib-0034] Nilsen, Ø A. , & Reiso, K. H. (2011). Scarring effects of unemployment. NHH Dept.of Economics Discussion Paper, 26.

[bjos13182-bib-0035] Paine, A. E. , McKay, S. , & Moro, D. (2013). Does volunteering improve employability? Insights from the british household panel survey and beyond. Voluntary Sector Review, 4(3), 355–376. 10.1332/204080513X13807974909244

[bjos13182-bib-0036] Petrovski, E. , Dencker‐Larsen, S. , & Holm, A. (2017). The effect of volunteer work on employability: A study with Danish survey and administrative register data. European Sociological Review, 33(3), 349–367. 10.1093/esr/jcx050

[bjos13182-bib-0037] Piatak, J. S. (2016). Time is on my side: A framework to examine when unemployed individuals volunteer. Nonprofit and Voluntary Sector Quarterly, 45(6), 1169–1190. 10.1177/0899764016628295

[bjos13182-bib-0038] Putnam, R. D. (2000). Bowling alone: America’s declining social capital. In Culture and politics: A reader (pp. 223–234). Palgrave Macmillan US.

[bjos13182-bib-0039] Qvist, H. Y. (2021). Hours of paid work and volunteering: Evidence from Danish panel data. Nonprofit and Voluntary Sector Quarterly, 50(5), 983–1008. 10.1177/0899764021991668

[bjos13182-bib-0040] Qvist, H. Y. (2022). Exploring the benefits of volunteering: Combining survey and administrative data in the nordic ‘laboratory’ (pp. 135–145). Policy Press. 10.56687/9781447356707-016

[bjos13182-bib-0041] Qvist, H. Y. , & Munk, M. D. (2018). The individual economic returns to volunteering in work life. European Sociological Review, 34(2), 198–210. 10.1093/esr/jcy004

[bjos13182-bib-0042] Rambøll Management Consulting . (2017). Frivillighedsundersøgelsen 2017: Metode rapport.

[bjos13182-bib-0043] Ravn, R. , & Nielsen, K. (2019). Employment effects of investments in public employment services for disadvantaged social assistance recipients. European Journal of Social Security, 21(1), 42–62. 10.1177/1388262719836797

[bjos13182-bib-0044] Ruiter, S. , & de Graaf, N. D. (2009). Socio‐economic payoffs of voluntary association involvement: A Dutch life course study. European Sociological Review, 25(4), 425–442. 10.1093/esr/jcn051

[bjos13182-bib-0045] Salamon, L. M. , & Anheier, H. K. (1992). In search of the non‐profit sector II: The problem of classification. Voluntas (Manchester, England), 3(3), 267–309. 10.1007/BF01397460

[bjos13182-bib-0046] Spence, M. (1973). Job market signaling. Quarterly Journal of Economics, 87(3), 355–374. 10.2307/1882010

[bjos13182-bib-0047] Spera, C. , Ghertner, R. , Nerino, A. , & DiTommaso, A. (2015). Out of work? Volunteers have higher odds of getting back to work. Nonprofit and Voluntary Sector Quarterly, 44(5), 886–907. 10.1177/0899764015605928

[bjos13182-bib-0048] Van Ingen, E. , & Kalmijn, M. (2010). Does voluntary association participation boost social resources? Social Science Quarterly, 91(2), 493–510. 10.1111/j.1540-6237.2010.00704.x

[bjos13182-bib-0049] Vooren, M. , Haelermans, C. , Groot, W. , & Maassen van den Brink, H. (2019). The effectiveness of active labor market policies: A meta‐analysis. Journal of Economic Surveys, 33(1), 125–149. 10.1111/joes.12269

[bjos13182-bib-0050] Wallrodt, S. , & Thieme, L. (2020). The role of sports volunteering as a signal in the job application process. European Sport Management Quarterly, 20(3), 255–275. 10.1080/16184742.2019.1598457

[bjos13182-bib-0051] Wiertz, D. , & Lim, C. (2019). The civic footprints of labor market participation: Longitudinal evidence from the United States, 2002–2015. Social Forces, 97(4), 1757–1784. 10.1093/sf/soy108

[bjos13182-bib-0052] Wilson, J. , & Musick, M. A. (1997). Work and volunteering: The long arm of the job. Social Forces, 76(1), 251–272. 10.2307/2580325

[bjos13182-bib-0053] Ziemek, S. (2006). Economic analysis of volunteers’ motivations—a cross‐country study. The Journal of Socio‐Economics, 35(3), 532–555. 10.1016/j.socec.2005.11.064

