# Supplementary material for: Lagging behind by doing good: How volunteering prolongs unemployment
Source: Br J Sociol. 2025 Jan 5;76(3):483–98. doi: 10.1111/1468-4446.13182 (PMC12163562; doi:10.1111/1468-4446.13182)

Online supplementary material to ”Lagging Behind by Doing Good: How Volunteering Prolongs Unemployment”

Table 1a. Lognormal accelerated failure time models predicting unemployment duration by volunteering (with interaction terms included).

|  | Model 1 | Model 2 | Model 3 |
| --- | --- | --- | --- |
| Volunteer | 0.071 | 0.183 | 0.376 |
|  | (0.296) | (0.182) | (0.377) |
| Unemployment benefits | -2.268^***^ | -2.206^***^ | -2.206^***^ |
|  | (0.170) | (0.148) | (0.148) |
| Labor market experience | -0.040 | -0.041 | -0.041 |
|  | (0.023) | (0.022) | (0.023) |
| Labor market experience × Labor market experience | 0.001^*^ | 0.001^*^ | 0.001^*^ |
|  | (0.001) | (0.001) | (0.001) |
| Unemployment history | 0.003 | 0.002 | 0.005 |
|  | (0.055) | (0.055) | (0.055) |
| Educational level (ref. = low) |  |  |  |
| Medium | -0.120 | -0.195 | -0.111 |
|  | (0.137) | (0.166) | (0.136) |
| High | -0.499^*^ | -0.474 | -0.490^*^ |
|  | (0.203) | (0.254) | (0.203) |
| Self-rated health (1-5) | -0.127 | -0.134 | -0.131 |
|  | (0.075) | (0.076) | (0.075) |
| Female | -0.086 | -0.091 | -0.090 |
|  | (0.123) | (0.123) | (0.123) |
| Immigrant background | -0.183 | -0.175 | -0.198 |
|  | (0.192) | (0.192) | (0.191) |
| Age | -0.001 | -0.001 | -0.000 |
|  | (0.009) | (0.009) | (0.010) |
| Volunteer × Unemployment benefits | 0.245 |  |  |
|  | (0.323) |  |  |
| Educational level (ref. = low) |  |  |  |
| Volunteer × Medium |  | 0.248 |  |
|  |  | (0.276) |  |
| Volunteer × High |  | -0.012 |  |
|  |  | (0.412) |  |
| Volunteer × Age |  |  | -0.003 |
|  |  |  | (0.011) |
| Constant | 3.994^***^ | 3.992^***^ | 3.937^***^ |
|  | (0.372) | (0.373) | (0.382) |
| Ln(σ) | 0.166^***^ | 0.165^***^ | 0.165^***^ |
|  | (0.033) | (0.033) | (0.033) |
| Ln(θ) | -1.313^***^ | -1.312^***^ | -1.311^***^ |
|  | (0.198) | (0.198) | (0.198) |
| Observations | 1027 | 1027 | 1027 |

Standard errors in parentheses

^*^ *p* < 0.05, ^**^ *p* < 0.01, ^***^ *p* < 0.001

Table 2a. Cox regression models predicting unemployment duration by volunteering

|  | Model 1 | Model 2 |
| --- | --- | --- |
| Volunteer | 0.808^**^ | 0.781^*^ |
|  | (0.063) | (0.081) |
| Unemployment benefits | 3.523^***^ | 4.421^***^ |
|  | (0.402) | (0.596) |
| Labor market experience | 1.032^*^ | 1.035 |
|  | (0.014) | (0.019) |
| Labor market experience × Labor market experience | 0.999^**^ | 0.999^*^ |
|  | (0.000) | (0.000) |
| Unemployment history | 0.961 | 0.971 |
|  | (0.031) | (0.043) |
| Educational level (ref. = low) |  |  |
| Medium | 1.138 | 1.115 |
|  | (0.094) | (0.122) |
| High | 1.247 | 1.362 |
|  | (0.143) | (0.218) |
| Self-rated health (1-5) | 1.094^*^ | 1.106 |
|  | (0.050) | (0.067) |
| Female | 1.049 | 1.070 |
|  | (0.077) | (0.105) |
| Immigrant background | 1.266^*^ | 1.281 |
|  | (0.150) | (0.201) |
| Age | 1.001 | 0.997 |
|  | (0.006) | (0.008) |
| θ |  | 0.310 |
|  |  | (0.060) |
| Observations | 1027 | 1027 |

Exponentiated coefficients; Standard errors in parentheses

^*^ *p* < 0.05, ^**^ *p* < 0.01, ^***^ *p* < 0.001

Table 3a. Cox regression models predicting unemployment duration by volunteering intensity

|  | Model 1 | Model 2 |
| --- | --- | --- |
| Monthly hours of volunteering (ref. = no volunteering) | 0.901 | 0.885 |
| 1-19 hours | (0.081) | (0.106) |
|  | 0.881 | 0.811 |
| 20 hours or more | (0.137) | (0.167) |
| Unemployment benefits | 3.512^***^ | 4.424^***^ |
|  | (0.401) | (0.599) |
| Labor market experience | 1.030^*^ | 1.034 |
|  | (0.014) | (0.019) |
| Labor market experience × Labor market experience | 0.999^**^ | 0.999^*^ |
|  | (0.000) | (0.000) |
| Unemployment history | 0.965 | 0.974 |
|  | (0.031) | (0.043) |
| Educational level (ref. = low) |  |  |
| Medium | 1.107 | 1.087 |
|  | (0.090) | (0.119) |
| High | 1.219 | 1.332 |
|  | (0.139) | (0.214) |
| Self-rated health (1-5) | 1.085 | 1.095 |
|  | (0.049) | (0.066) |
| Female | 1.053 | 1.077 |
|  | (0.078) | (0.106) |
| Immigrant background | 1.284^*^ | 1.285 |
|  | (0.152) | (0.203) |
| Age | 1.001 | 0.997 |
|  | (0.006) | (0.008) |
| θ |  | 0.319 |
|  |  | (0.061) |
| Observations | 1027 | 1027 |

Exponentiated coefficients; Standard errors in parentheses

^*^ *p* < 0.05, ^**^ *p* < 0.01, ^***^ *p* < 0.001

Table 4a. Lognormal accelerated failure time models predicting unemployment duration by volunteering (only first unemployment spell for each individual).

|  | Model 1 | Model 2 |
| --- | --- | --- |
| Volunteer | 0.117 | 0.370^*^ |
|  | (0.185) | (0.164) |
| Unemployment benefits |  | -1.602^***^ |
|  |  | (0.181) |
| Labor market experience |  | -0.039 |
|  |  | (0.027) |
| Labor market experience # Labor market experience |  | 0.001 |
|  |  | (0.001) |
| Unemployment history |  | 0.063 |
|  |  | (0.067) |
| Medium |  | -0.209 |
|  |  | (0.174) |
| High |  | -0.163 |
|  |  | (0.259) |
| Self-rated health (1-5) |  | -0.112 |
|  |  | (0.091) |
| Female=1 |  | -0.031 |
|  |  | (0.154) |
| Immigrant background=1 |  | -0.220 |
|  |  | (0.249) |
| Age |  | 0.003 |
|  |  | (0.012) |
| Constant | 2.131^***^ | 3.841^***^ |
|  | (0.103) | (0.467) |
| Ln(σ) | 0.388^***^ | 0.262^***^ |
|  | (0.033) | (0.041) |
| Observations | 437 | 437 |

Standard errors in parentheses

^*^ *p* < 0.05, ^**^ *p* < 0.01, ^***^ *p* < 0.001

Figure 1a: Conditional marginal effects of volunteering on unemployment durations in weeks and calculated with respect to the predicted median of unemployment durations.


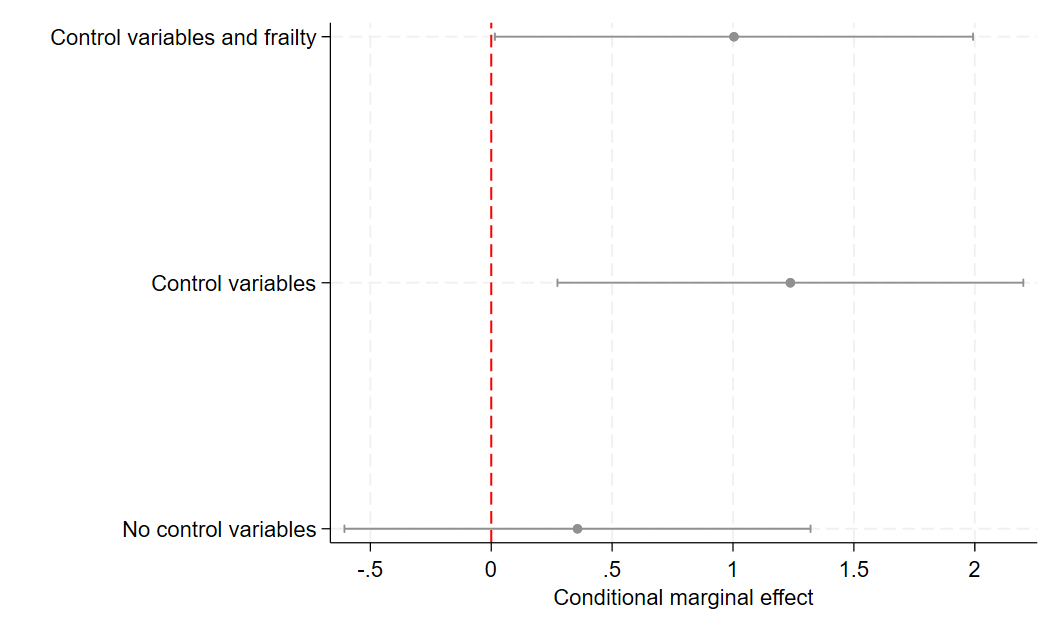

Supplement: Supplementary file 1 — Supporting Information S1 [file BJOS-76-483-s001.docx]
